# Chronic whipworm infection exacerbates *Schistosoma mansoni* egg-induced hepatopathology in non-human primates

**DOI:** 10.1186/s13071-020-3980-z

**Published:** 2020-02-28

**Authors:** Loc Le, Sabiha Khatoon, Paola Jiménez, Christopher Peterson, Rebecca Kernen, Weidong Zhang, Adebayo J. Molehin, Samra Lazarus, Justin Sudduth, Jordan May, Souvik Karmakar, Juan U. Rojo, Gul Ahmad, Workineh Torben, David Carey, Roman F. Wolf, James F. Papin, Afzal A. Siddiqui

**Affiliations:** 1 Center for Tropical Medicine and Infectious Diseases, Texas Tech University Health Sciences Center, Lubbock, TX USA; 2 Department of Internal Medicine, School of Medicine, Texas Tech University Health Sciences Center, Lubbock, TX USA; 3 Department of Veterinary Pathobiology, College of Veterinary Medicine, Texas A&M University, College Station, TX USA; 4 Center for Cancer Research, National Cancer Institute, National Institutes of Health, Bethesda, MD USA; 5 Department of Molecular, Cellular and Biomedical Sciences, University of New Hampshire, Durham, NH USA; 6 Department of Natural Sciences, Peru State College, Peru, NE USA; 7 Department of Biological Sciences, Louisiana State University, Alexandria, LA USA; 8 Department of Pathology, University of Oklahoma Health Sciences Center, Oklahoma City, OK USA; 9 Oklahoma City VA Health Care System, Oklahoma City, OK USA

**Keywords:** Schistosomiasis, Trichuriasis, Helminths, Co-infection, NTDs, Hepatopathology

## Abstract

**Background:**

Schistosomiasis continues to inflict significant morbidity and mortality in the tropical and subtropical regions of the world. The disease endemicity overlaps with the transmission of other parasitic diseases. Despite the ubiquity of polyparasitism in tropical regions, particularly in rural communities, little is known about the impact of multiple helminth infections on disease progression. In this pilot study, we describe the influence of chronic *Trichuris trichiura* infection on *Schistosoma mansoni* egg-induced hepatopathology in infected baboons.

**Methods:**

Baboons with or without underlying whipworm infection were challenged with *S. mansoni* cercariae to establish schistosomiasis. Adult *S. mansoni* worms were recovered by perfusion and enumerated, hepatic granulomas were quantified *via* light microscopy, and transcriptional profiling of tissues were completed using RNA sequencing technologies.

**Results:**

Co-infection with both *S. mansoni* and *T. trichiura* resulted in higher female schistosome worm burden and significantly larger liver granuloma sizes. Systems biology analyses of peripheral blood mononuclear cells (PBMC) revealed pathways associated with increased liver damage in co-infected baboons.

**Conclusions:**

Underlying chronic whipworm infection intensified schistosome egg-induced liver pathology in infected baboons. RNA-Seq analysis provided insight into pathways associated with increased liver damage, corroborating histological findings.
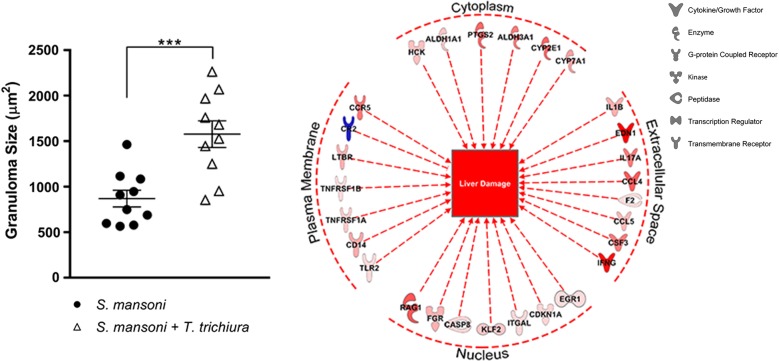

## Background

Neglected tropical diseases (NTDs) are a group of diseases that disproportionately affect communities of poverty. The lack of adequate infrastructure and public sanitation in these communities contribute to maintaining the vicious cycle of chronic disease burden and poverty despite current efforts to control and interrupt transmission using mass drug administration. Currently, 252 million people are infected with schistosomiasis, an estimate that continues to rise as diagnostic methodologies become more refined [1]. Chronic intestinal schistosome infection can result in hepatosplenomegaly and eventually death due to internal bleeding. It is not uncommon for the same populations to be infected or be at risk for infection with soil-transmitted helminths (STH) [2]. Over 465 million people currently live with whipworm infection [1]. Chronic whipworm, or *Trichuris trichiura*, infection can result in abdominal pain, anemia, and wasting, particularly in children. Anemia and *Trichuris* Dysentery Syndrome contribute to protein energy malnutrition which leads to an immunocompromised state that can exacerbate subsequent helminth infections [3, 4]. As major sources of morbidity and disability rather than mortality, the disability-adjusted life years attributed to schistosomiasis and trichuriasis are 3.31 million/year and 0.64 million/year, respectively [1].

There are insufficient studies on the interactions of trichuriasis and schistosomiasis, specifically on the influence of co-infection on *Schistosoma mansoni* egg-induced hepatopathology. Others have found that concomitant infections of *Schistosoma japonicum* and *Trichuris* significantly increased the odds of anemia in children [4] and that mice with established chronic *Trichuris muris* infection and challenged with *S. mansoni* developed significantly higher *S. mansoni* worm burden and egg and granuloma burden in the liver [5]. To prepare for deployment of interventions such as vaccines against schistosomiasis [6], it is critical to understand the interactions between potential confounding factors that will be present in endemic areas, such as polyparasitism. This present study aimed to evaluate whether concurrent infection with *T. trichiura* could influence *S. mansoni* egg-induced hepatopathology using the non-human primate model (baboons). Baboons develop a human-like acute schistosomiasis syndrome after exposure to the cercariae and chronic hepatic/intestinal clinical manifestations. Herein, we describe the first transcription profiling using next-generation sequencing for liver damage in baboons infected with schistosomiasis and trichuriasis, indicating several pathways by which co-infection may exacerbate hepatopathology.

## Methods

### Animals

Male and female olive baboons (*Papio anubis*) aged 2 to 4 years-old were obtained from the University of Oklahoma Health Sciences Center (OUHSC) and were housed in facilities accredited by the AAALAC.

*Schistosoma mansoni*-infected *Biomphalaria glabrata* (Puerto Rican strain) snails were obtained from the Schistosomiasis Resource Center, Biomedical Research Institute (Rockville, MD, USA).

### Parasite challenge and necropsy

We selected baboons that had prior exposure to *T. trichiura* for the co-infection group (*n* = 10) and baboons that had no prior exposure to *T. trichiura* for the single infection group (*n* = 10) as identified by microscopic identification of *T. trichiura* eggs in stool samples. Baboon colonies commonly harbor *T. trichiura* and, in those cases, deworming with anthelmintics is routine. In this study, baboons selected for the co-infection group did not undergo deworming and specific-pathogen-free baboons were utilized for the single infection group. We subsequently exposed all baboons to 1000 *S. mansoni* cercariae percutaneously as previously described [7]. Eight weeks after infection with *S. mansoni*, necropsies were performed. Briefly, the hepatic portal system and mesenteric vasculature were perfused to recover adult schistosome worms. Blood was collected for PBMC isolation by density gradient centrifugation using HISTOPAQUE-1077 (Sigma-Aldrich, St. Louis, Missouri, USA) and stored in freezing media (10% DMSO in fetal bovine sera and RPMI) until later use for RNA-purification. Liver samples were collected for tissue egg burden assessment and granuloma quantification *via* histology. For determination of schistosome egg burden in tissue, liver samples were digested overnight in 4% potassium hydroxide at 37 °C without CO_2_. This suspension was then washed and resuspended with a solution of 1.2% (w/v) NaCl and eggs were enumerated under light microscopy to determine the number of eggs per gram of tissue [7]. Excised spleen samples and mesenteric lymph nodes were mashed through nylon cell strainers and stored in freezing media until RNA purification for RNA-sequencing.

### Histology

Sections of liver were fixed in 10% neutral-buffered formalin, dehydrated in ascending grades of alcohol, and embedded in paraffin. Paraffin blocks were cut into 5-micron sections and processed for staining with hematoxylin and eosin. Stained slides were observed *via* light microscopy at 100× magnification for granuloma quantification as previously described [8]. The diameter of each granuloma was measured *via* a straight line bisecting the central egg and the area of each granuloma was calculated assuming an area of a circle (Additional file 1: Figure S1).

### RNA purification

Total RNA was isolated from PBMCs, splenocytes, and mesenteric lymph nodes of each animal using GenElute™ mammalian total RNA miniprep kit (Millipore Sigma, St. Louis, MO, USA) as previously described [7]. Total RNA concentrations were measured using the Qubit® 3.0 Fluorometer and RNA HS assay kit (Thermo Fisher Scientific, Waltham, MA, USA). RNA quality was assessed using Agilent 2200 TapStation (Agilent, Santa Clara, CA).

### RNA sequencing and pathway analysis

Total RNA from PBMCs, spleen, and mesenteric lymph node cells were used to prepare the libraries as previously described [7]. RNA sequencing was performed using the Illumina-HiSeq 2500 platform. Raw sequence reads containing base call information were demultiplexed using bcl2fastq software and the quality of sequencing was evaluated using FastQC software (Babraham Bioinformatics). Quality filtered reads for each animal from each tissue sample were mapped to the *Homo sapiens* genome (GRCh37) using QSeq® version 15.0 software (DNASTAR, Madison, WI, USA) for differential gene expression analysis using RPKM normalization. Differential expression was considered significant at *P* < 0.05 (Student’s t-test and the Benjamin Hochberg false discovery rate method) with a cut-off of 2-fold change.

Ingenuity Pathway Analysis (IPA) (Qiagen, Venlo, Netherlands) was utilized to generate pathway analyses. Fisher’s exact test (right-tailored) was used to calculate *P*-values, and activation *Z*-score was used to predict activation or inhibition of a process or the directional effect of gene expression. Data analysis and plots were generated using GraphPad Prism v7 (GraphPad Software, La Jolla, CA).

Selected genes were validated *via* quantitative real-time PCR as previously described [7] (Additional file 1: Table S1). Primers for qRT-PCR were designed from mRNA sequences obtained from the NCBI for *Papio anubis* genes in order to see their expression using qRT-PCR. The list of primer sequences used for qRT-PCR is provided in Additional file 1: Table S2. Briefly, total RNA from PBMCs was extracted using GenElute™ Mammalian Miniprep kit (Sigma-Aldrich, St. Louis, MO, USA) and first strand cDNA synthesis was completed using the Maxima First Strand cDNA synthesis kit (Thermo Fisher Scientific). PCR amplification of selected genes was carried out using SYBR Premix Ex Taq™ (TIi RNase H Plus; Takara, Japan) on a StepOne™ plus Real-time PCR system (Thermo Fisher Scientific). All reactions were carried out in triplicates and results analyzed using DataAssist™ software v3.0 (Thermo Fisher Scientific).

### Statistical analysis

For statistical comparison between co-infected animals and animals infected with *S. mansoni* alone, one- or two-way analysis of variance (ANOVA), and/or two-tailed Student’s t-test were performed, and statistical significance was determined at the 95% confidence interval (*P* < 0.05).

## Results

### Parasitological endpoints are distinct in baboons infected with both *S. mansoni* and *T. trichiura* compared to *S. mansoni* alone

**Fig. 1:**
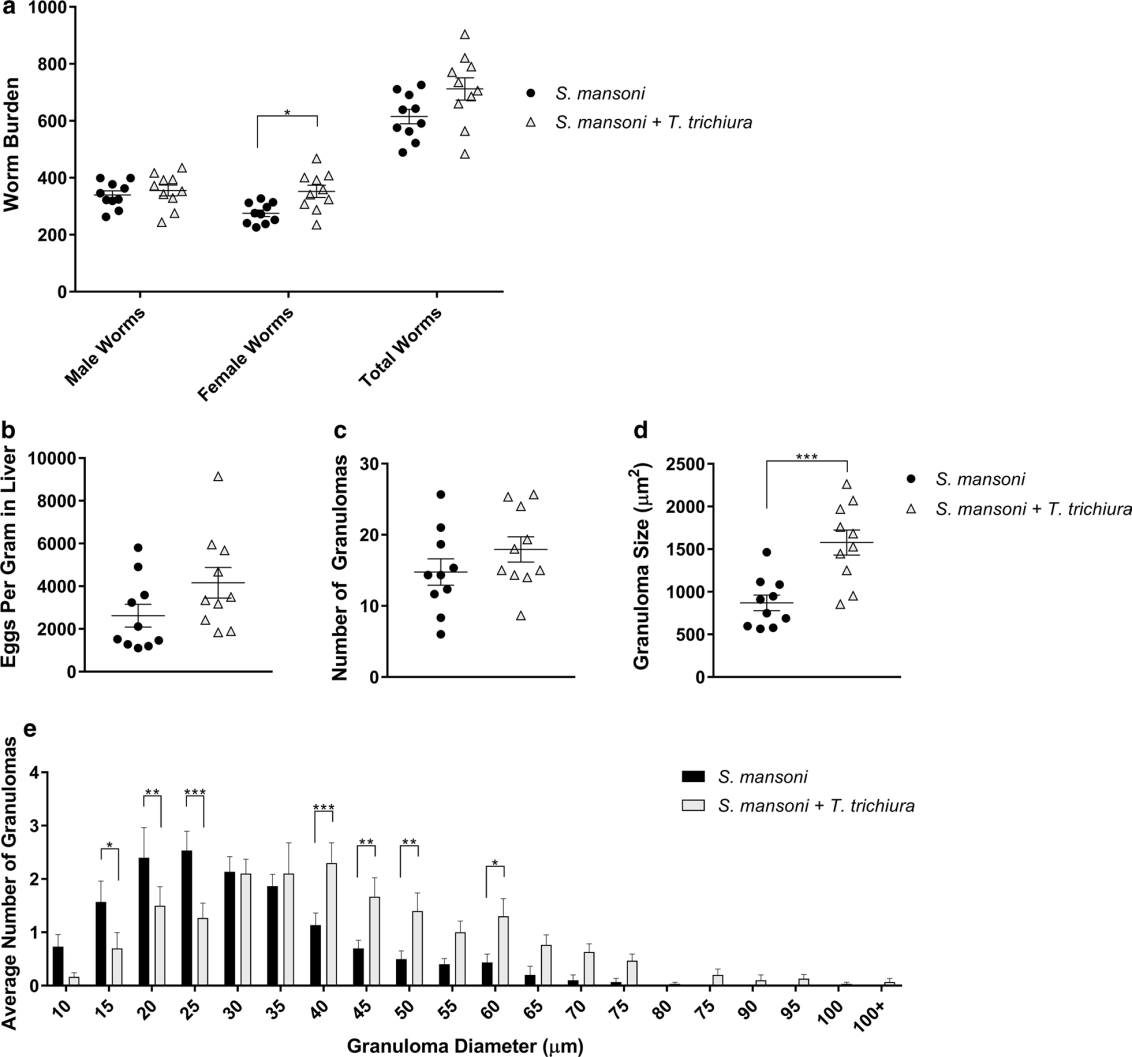
Parasitological endpoints of schistosomiasis in animals infected with *S. mansoni* alone or a combination of *S. mansoni* and *T. trichiura*. Baboons were obtained from OUHSC with or without pre-existing *T. trichiura* infection. All baboons were subsequently infected with 1000 *S. mansoni* cercariae and sacrificed after 8 weeks. **a** *S. mansoni* worm burden. **b** Eggs per gram in liver tissue. **c** Average number of granulomas in the liver within a 1 × 1 cm^2^ area. **d** Average size of granulomas in the liver within a 1 × 1 cm^2^ area. **e** Number and size of granulomas within a 1 × 1 cm^2^ area. Error bars represent the mean and standard error of the mean. *P < 0.05, **P < 0.01, ***P < 0.001

In endemic areas, the first contact with cercariae-contaminated water takes place in early childhood while contact with eggs from STH can occur within the first year of life [9]. As such, we selected baboons that had prior exposure to *T. trichiura* for the co-infection group and baboons that had no prior exposure to *T. trichiura*. We exposed all baboons to 1000 *S. mansoni* cercariae as previously described [7]. When we compared the worm burden of baboons infected with *S. mansoni* and *T. trichiura*, henceforth considered the co-infected group, to baboons infected with *S. mansoni* alone, we found 27.9% (*t*_(18)_ = 3.1747, *P* = 0.0052) more female worms (Fig. 1a). The number of male worms and total worm burden were not significantly different between the groups. Liver tissue egg burden quantification revealed no significant difference; schistosome infection alone resulted in an average of 2620 ± 1687 eggs per gram of liver tissue compared to co-infected animals with an average of 4163 ± 2268 eggs per gram of liver tissue (*t*_(18)_ = 1.7270, *P* = 0.1013) (Fig. 1b).

Systematic analysis of granulomas from liver sections revealed that average number of granulomas in a 1 cm^2^ area between both groups were not significant (Fig. 1c). However, in comparing the average granuloma size of co-infected baboons, we found that granulomas were nearly double in size compared to those found in animals infected with *S. mansoni* alone (*t*_(18)_ = 4.0765, *P* = 0.0007) (Fig. 1d). Co-infected animals had an average granuloma size of 1578 ± 465 µm^2^ compared to animals infected with *S. mansoni* alone with an average granuloma size of 870 ± 292 µm^2^. In summary, the average size of granulomas observed were significantly larger in baboons infected with both parasites when compared to baboons infected with *S. mansoni* alone (Fig. 1e).

### Whole transcriptome sequencing shows distinct transcriptional profiles that indicate greater liver damage in baboons co-infected with *S. mansoni* and *T. trichiura*

**Fig. 2:**
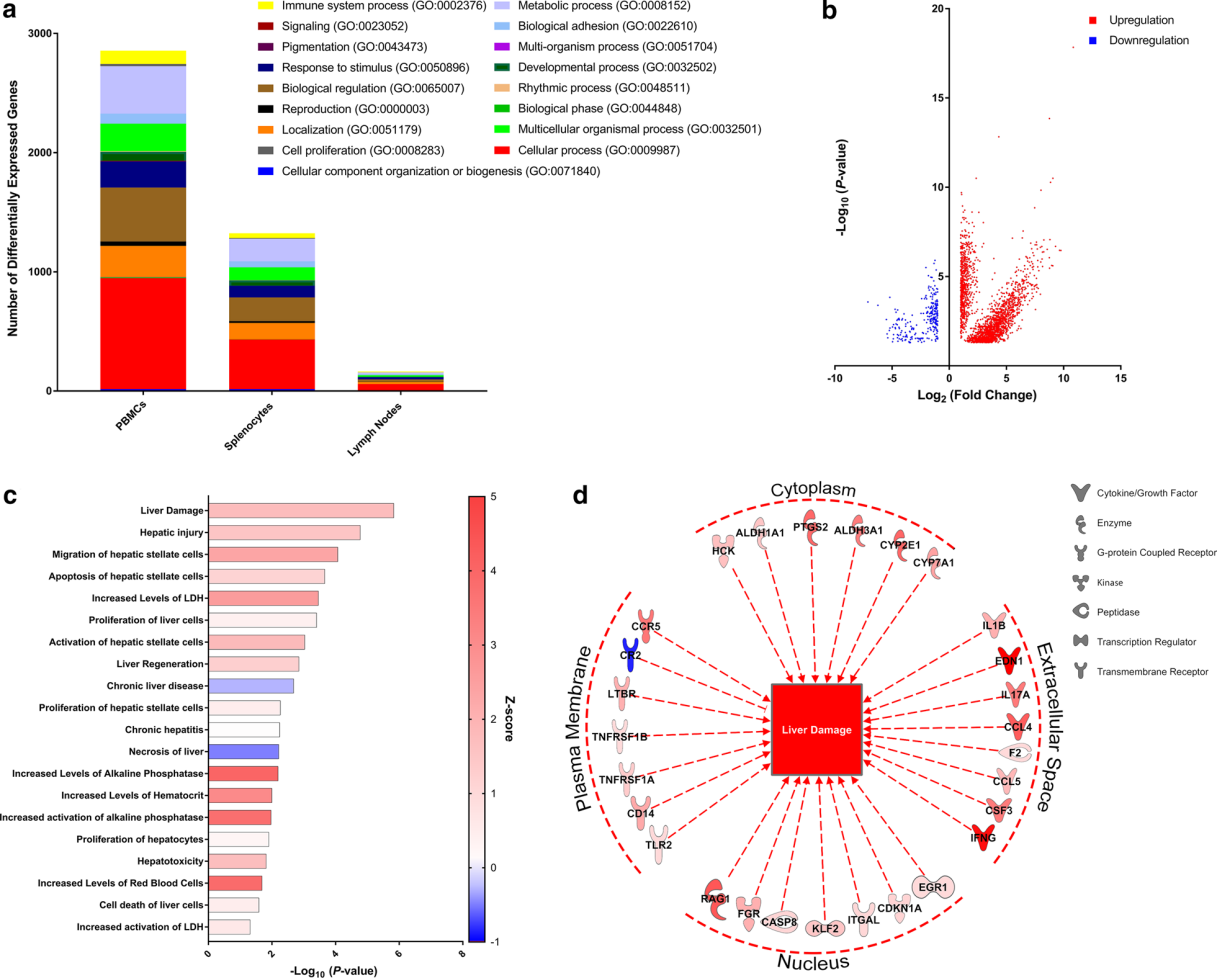
RNA-Seq analysis reveal distinct transcriptional profiles that indicate greater liver damage in animals co-infected with *S. mansoni* and *T. trichiura*. **a** Distribution of differentially expressed genes in baboons co-infected with *S. mansoni* and *T. trichiura* compared to *S. mansoni* alone. **b** Significantly upregulated or downregulated genes in PBMCs. Each colored dot represents one gene. **c** Disease pathway analyses on generated using IPA for PBMCs. Bars are plotted based on the − log_10_(*P*-value) and colored red denoting upregulation/activation and blue representing downregulation/inhibition according to the *Z*-score, a prediction of activation or inhibition based on the degree of overlap between directional expression of genes from the observed data and the Qiagen-curated public database. **d** Schematic representation of genes from PBMCs involved in activation of liver damage pathway. Red represents upregulation and blue represents downregulation. For all comparisons, the threshold for statistical significance was *P* < 0.05

To understand the transcriptional alterations elicited by preexisting *T. trichiura* infection on schistosomiasis, we performed RNA-Seq on peripheral blood mononuclear cells (PBMCs), splenocytes, and mesenteric lymph nodes. Based on a *P*-value < 0.05, 2930 genes were differentially expressed in PBMCs, 1805 genes in splenocytes, and 90 genes in mesenteric lymph nodes. Differential expression in these genes reflect changes in many biological processes including cellular processes, cell proliferation, and response to stimulus (Fig. 2a). Focusing on differentially expressed genes in PBMCs, we observed 2634 genes that were significantly upregulated and 298 significantly downregulated genes when comparing the co-infected group compared to animals infected with only *S. mansoni* (Fig. 2b).

Pathways and functional analysis on PBMCs depicted signatures of liver damage, corroborating the data on liver granulomas described earlier. Indeed, pathways predicting liver damage (*P* = 0.000001) and hepatic injury (*P* = 0.00002) were among the most significant (Fig. 2c). Several significantly upregulated genes were predicted to lead to activation of liver damage and hepatic injury, including prostaglandin-endoperoxide synthase 2 (PTGS2), cytochrome P450 family 2 subfamily E member 1 (CYP2E1), cytochrome P450 family 7 subfamily A member (CYP7A1), aldehyde dehydrogenase 1 family member A1 (ALDH1A1), aldehyde dehydrogenase 3 family member A1 (ALDH3A1), and others (Fig. 2d). Interestingly, the pathways predicted to be inhibited include chronic liver disease and necrosis of the liver. Upregulation in growth factors like epidermal growth factor (EGF), heparin binding EGF like growth factor (HBEGF), inhibin subunit alpha (INHA), nerve growth factor receptor (NGFR), and others were predicted to contribute to the inhibition of necrosis of the liver, possibly in response to ongoing liver damage cause by chronic schistosomiasis and trichuriasis.

## Discussion

Schistosomiasis and trichuriasis continue to be major contributors to the global disease burden, often within the same communities. Taken together, our work demonstrates that underlying whipworm infection exacerbates *S. mansoni* egg-induced hepatopathology. Co-infection with *T. trichiura* and *S. mansoni* resulted in significantly higher female schistosome worm burden compared to infection with *S. mansoni* alone. Interestingly, while the number of granulomas quantified in the livers of both groups did not differ significantly, coinciding with the liver egg counts, the average size of granulomas was nearly double in the co-infected group (1578 ± 465 µm^2^) compared to animals with only schistosomiasis (870 ± 292 µm^2^).

Whole transcriptome analysis of PBMCs provided insight into how gene expression correlated with the significant increase in average granuloma size. Disease pathways including liver damage, hepatic injury, migration of hepatic stellate cells, apoptosis of hepatic stellate cells, increased levels of LDH, and others were predicted to be activated. Several differentially expressed genes were common between these pathways, including cytochromes (CYP2E1 and CYP7A1), cyclooxygenase (PTGS2), aldehyde dehydrogenases (ALDH1A1 and ALDH3A1), and chemokines and cytokines (IFNγ, CCL4, CCL5, IL1β, IL17A). Although splenocytes and lymph nodes were assayed in this study, we did not have enough differentially expressed genes to draw definitive conclusions. For example, while we observed predicted activation of similar pathways in lymph node samples such as liver damage (*Z*-score: 0.916) and hepatic injury (*Z*-score: 0.873), fewer differentially expressed genes played a role in those predictions (7 genes for liver damage and 5 genes for hepatic injury) compared to the 60–80 genes differentially expressed in the PBMCs for the same pathways. Common differentially expressed genes in the PBMCs, spleen samples, and lymph nodes that predict activation of liver damage and hepatic injury include CCL4, CCL5, CCR5, IFNγ, and PTGS2.

The activity of the cytochrome P450 family of enzymes, which include CYP2E1 and CYP7A1, has been reported to be modulated by intestinal schistosomiasis, contingent on granulomatous reactions around the eggs in the tissue [10]. Chronic infection with *S. mansoni* in mice coincided with decreased levels of liver cytochrome P450 which correlate with fibrosis and progression of hepatopathology. Surprisingly, we observed increased levels of cytochrome P450 due to co-infection compared to infection with *S. mansoni* alone. Activation of the liver damage pathway, in this case, may be due to the generation of reactive oxygen species and toxic metabolites by cytochrome P450 during infection [11]. Another gene that was predicted to activate the liver damage pathway was prostaglandin-endoperoxide synthase 2 (PTGS2), also known as COX2. It has been shown that COX2 can be induced by *S. mansoni* to downregulate IL-10-dependent host immune responses in the skin of mice [12]; co-infection resulted in higher COX2 expression which potentially down-regulates immune responses in the liver, thus activating the liver damage pathway.

Upregulation of ALDH1A1 and ALDH3A1 are predicted to lead to the activation of the liver damage pathway. Aldehyde dehydrogenases (ALDH) and Vitamin A play a role in reducing the pathogenic effects of infection [13] and it has been demonstrated that infection with *Trichuris muris* in mice reduces ALDH [14]. In contrast, others have shown that upregulation of aldehyde dehydrogenases, also known as retinal dehydrogenases, are induced during a retinoid-dependent type-2 immune response to chronic infection with *S. mansoni* [15]. It is likely that co-infection with *T. trichiura* and *S. mansoni* in our study resulted in a stronger response to the schistosomiasis rather than trichuriasis within the co-infected group, thus driving Th2 responses, possibly in part due to retinoic acid catalyzed by ALDH [15]. In subsequent studies, we plan to measure liver enzymes such as AST and ALT to corroborate the liver damage predicted by differential gene expression from RNA-seq. Although genes related to immune function were differentially expressed, pathway analysis using IPA was unable to predict the role of canonical immune pathways such as the Th1 or Th2 pathway (*Z*-scores: NaN). Further studies are required to describe the nuanced transcriptional gene expressions that comprise the immune response to co-infection with *S. mansoni* and *T. trichiura*, with insights into how a vaccine against schistosomiasis could mediate protection for animals with multiple helminth infections.

## Conclusions

In conclusion, RNA-Seq analysis provided insight into the various pathways by which liver damage is exacerbated in baboons co-infected with *T. trichiura* and *S. mansoni* compared to infection with *S. mansoni* alone, supporting the histological analysis of liver granulomas.

## Supplementary information

**Additional file 1: Figure S1.** Representative liver granuloma measurement. **Table S1.** qRT-PCR validation of genes. **Table S2.** List of primer sequences used.

## Data Availability

All data generated or analyzed during this study are included in this published article and its additional files.

## References

[1] Hotez PJ, Alvarado M, Basanez MG, Bolliger I, Bourne R, Boussinesq M (2014). The global burden of disease study 2010: interpretation and implications for the neglected tropical diseases. PLoS Negl Trop Dis..

[2] Brooker S, Miguel EA, Moulin S, Luoba AI, Bundy DA, Kremer M (2000). Epidemiology of single and multiple species of helminth infections among school children in Busia District, Kenya. East Afr Med J..

[3] Robertson LJ, Crompton DW, Sanjur D, Nesheim MC (1992). Haemoglobin concentrations and concomitant infections of hookworm and *Trichuris trichiura* in Panamanian primary schoolchildren. Trans R Soc Trop Med Hyg..

[4] Ezeamama AE, McGarvey ST, Acosta LP, Zierler S, Manalo DL, Wu HW (2008). The synergistic effect of concomitant schistosomiasis, hookworm, and trichuris infections on childrenʼs anemia burden. PLoS Negl Trop Dis..

[5] Bickle QD, Solum J, Helmby H (2008). Chronic intestinal nematode infection exacerbates experimental *Schistosoma mansoni* infection. Infect Immun..

[6] Siddiqui AA, Siddiqui SZ (2017). Sm-p80-based schistosomiasis vaccine: preparation for human clinical trials. Trends Parasitol..

[7] Zhang W, Molehin AJ, Rojo JU, Sudduth J, Ganapathy PK, Kim E (2018). Sm-p80-based schistosomiasis vaccine: double-blind preclinical trial in baboons demonstrates comprehensive prophylactic and parasite transmission-blocking efficacy. Ann N Y Acad Sci..

[8] Le L, Molehin AJ, Nash S, Sennoune SR, Ahmad G, Torben W (2018). *Schistosoma* egg-induced liver pathology resolution by Sm-p80-based schistosomiasis vaccine in baboons. Pathology..

[9] Drake LJ, Bundy DA (2001). Multiple helminth infections in children: impact and control. Parasitology..

[10] Conte FP, Fidalgo-Neto AA, Manhaes-Rocha DA, Paumgartten FJ, De-Oliveira AC (2007). Activity of liver microsomal enzymes during the chronic phase of murine schistosomiasis. Braz J Med Biol Res..

[11] Stavropoulou E, Pircalabioru GG, Bezirtzoglou E (2018). The role of cytochromes P450 in infection. Front Immunol..

[12] Ramaswamy K, Kumar P, He YX (2000). A role for parasite-induced PGE2 in IL-10-mediated host immunoregulation by skin stage schistosomula of *Schistosoma mansoni*. J Immunol..

[13] Sommer A (2008). Vitamin a deficiency and clinical disease: an historical overview. J Nutr..

[14] Hurst RJ, Else KJ (2013). The retinoic acid-producing capacity of gut dendritic cells and macrophages is reduced during persistent *T muris* infection. Parasite Immunol..

[15] Broadhurst MJ, Leung JM, Lim KC, Girgis NM, Gundra UM, Fallon PG (2012). Upregulation of retinal dehydrogenase 2 in alternatively activated macrophages during retinoid-dependent type-2 immunity to helminth infection in mice. PLoS Pathog..

