# Supplementary material for: Chronic whipworm infection exacerbates Schistosoma mansoni egg-induced hepatopathology in non-human primates
Source: Parasit Vectors. 2020 Feb 28;13:109. doi: 10.1186/s13071-020-3980-z (PMC7048111; doi:10.1186/s13071-020-3980-z)
Supplement: Supplementary file 1 — Additional file 1: Figure S1. Representative liver granuloma measurement. Table S1. qRT-PCR validation of genes. Table S2. List of primer sequences used. [file 13071_2020_3980_MOESM1_ESM.docx]

**Additional file 1: Figure S1.** Representative Liver Granuloma Measurement.


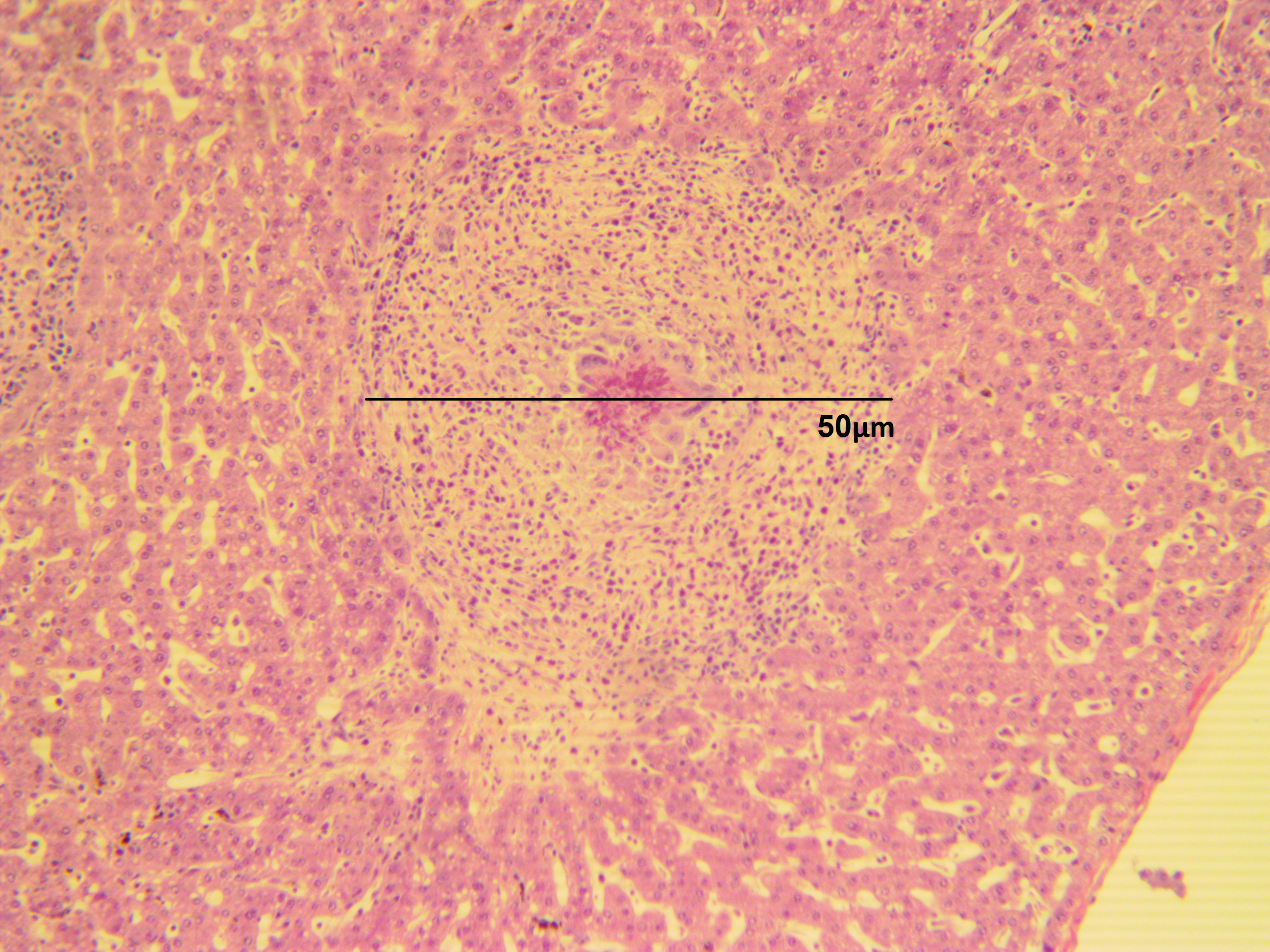


**Additional file 1: Table S1.** qPCR Validation of Genes.

| **DEGs** | **Log2 fold change (RNA-seq)** | **Log2 fold change (qRT-PCR)** |
| --- | --- | --- |
| CSF3 | 3.6 | 0.86715 |
| CYP2E1 | 4.19054 | 3.77376 |
| CYP7A1 | 2.56064 | 0.44159 |
| EDN1 | 7.06418 | 1.8245 |
| EGR1 | 1.00709 | 0.9266 |
| IFNγ | 6.62238 | 1.08195 |
| IL12A | 9.06282 | 3.0628 |
| IL8 | 3.05628 | 1.37762 |
| PTGS2 | 4.15896 | 0.97722 |
| TNFB | 1.01056 | -0.4183 |

**Additional file 1: Table S2.** List of primer sequences used.

| CSF3 | Sense 5’ gaagctgtgtgccacctaca 3’ |
| --- | --- |
|  | Antisense 5’ tagaggaagaggccgctatg 3’ |
| CYP2E1 | Sense 5 ’acccgagacaccattttcag 3’ |
|  | Antisense 5’ tccagcacacactcgttttc 3’ |
| CYP7A1 | Sense 5’ caccttgaggacggttccta 3’ |
|  | Antisense 5’cgatccaaagggcatgtagt 3’ |
| EDN1 | Sense 5’ gctcgtccctgatggataaa 3’ |
|  | Antisense 5’ tgtctttggccctgagttct 3’ |
| EGR1 | Sense 5’ agctggaggagatgatgctg 3’ |
|  | Antisense 5’ gaaaagactctgcggtcagg 3’ |
| GAPDH | Sense 5’ aacatcatccctgcctctactg 3’ |
|  | Antisense 5’ ttggcaggtttttccagacg 3’ |
| IFNγ | Sense 5’ gtggagaccatcaaggaagac 3’ |
|  | Antisense 5’ tgtactgctttgcgttggac 3’ |
| IL8 | Sense 5’ accacactgcgtcaatacag 3’ |
|  | Antisense 5’aacttctccacaaccctctgc 3’ |
| IL12a | Sense 5’ accaggtggagttcaagacca 3’ |
|  | Antisense 5’ gcccgaattctgaaagcatg 3’ |
| PTGS2 | Sense 5’ gccatctttggtgagaccat 3’ |
|  | Antisense 5’ gaaaacccacttctccacca 3’ |
| TNFRSF1B | Sense 5’ gaccaggtggaaactcaagc 3’ |
|  | Antisense 5’ tgcaaatatccgtggatgaa 3’ |
